# Mass spectrometry-based proteomics as a tool to identify biological matrices in forensic science

**DOI:** 10.1007/s00414-012-0747-x

**Published:** 2012-07-29

**Authors:** Katleen Van Steendam, Marlies De Ceuleneer, Maarten Dhaenens, David Van Hoofstat, Dieter Deforce

**Affiliations:** Laboratory for Pharmaceutical Biotechnology, Ghent University, Harelbekestraat 72, 9000 Ghent, Belgium

**Keywords:** Mass spectrometry, Species identification, Biological matrices, Proteomics

## Abstract

In forensic casework analysis, identification of the biological matrix and the species of a forensic trace, preferably without loss of DNA, is of major importance. The biological matrices that can be encountered in a forensic context are blood (human or non-human), saliva, semen, vaginal fluid, and to a lesser extent nasal secretions, feces, and urine. All these matrices were applied on swabs and digested with trypsin in order to obtain peptides. These peptides were injected on a mass spectrometer (ESI Q-TOF) resulting in the detection of several biomarkers that were used to build a decision tree for matrix identification. Saliva and blood were characterized by the presence of alpha-amylase 1 and hemoglobin, respectively. In vaginal fluid, cornulin, cornifin, and/or involucrin were found as biomarkers while semenogelin, prostate-specific antigen, and/or acid phosphatase were characteristic proteins for semen. Uromodulin or AMBP protein imply the presence of urine, while plunc protein is present in nasal secretions. Feces could be determined by the presence of immunoglobulins without hemoglobin. The biomarkers for the most frequently encountered biological matrices (saliva, blood, vaginal fluid, and semen) were validated in blind experiments and on real forensic samples. Additionally, by means of this proteomic approach, species identification was possible. This approach has the advantage that the analysis is performed on the first “washing” step of the chelex DNA extraction, a solution which is normally discarded, and that one single test is sufficient to determine the identity and the species of the biological matrix, while the conventional methods require cascade testing. This technique can be considered as a useful additional tool for biological matrix identification in forensic science and holds the promise of further automation.

**Electronic supplementary material:**

The online version of this article (doi:10.1007/s00414-012-0747-x) contains supplementary material, which is available to authorized users.

## Introduction

In forensic science, DNA typing and fingerprint analysis are the most prominent means for identifying individuals involved in a crime. However, determining the biological origin of a trace can be equally important in reconstructing the events that took place. For example, when a suspect accused of unwanted internal groping denies charges, the presence of vaginal fluid under his fingernails can help in pinpointing the suspect.

The biological matrices that are most often found at a crime scene are blood, semen, vaginal fluid, and saliva. Less frequently, other matrices such as nasal secretion, urine, and feces can also be found. Biochemical tests that can determine whether biological fluids are present and that can identify the matrix are already available. For example, blood is detected by means of luminol or benzidine [1, 2]. For semen, forensic tests currently focus on semenogelin, prostatic acid phosphatase, and prostate-specific antigen (PSA) (RSID-Semen test, Phosphatesmo Km Paper, and Seratec PSA semiquant, respectively) [3–7]. The acid phosphatase test, however, is an indirect test measuring enzyme activity. When these tests were compared, PSA detection (Seratec PSA semiquant test) gave the best results with constant satisfactory sensitivity over time [5]. Alpha-amylase tests such as the Phadebas assay are currently available for the detection of saliva. The latter is based on Bio-Degradable Starch Microspheres which are covalently bound to a blue dye. When alpha-amylase is present in a sample, the dye is released [8]. However, similar to the acid phosphatase test, the Phadebas assay is an indirect test that measures the activity of amylase. Therefore, these biochemical tests sometimes lack specificity because they cannot differentiate between the alpha-amylase 1 (present in saliva) and alpha-amylase 2 (present in semen and vaginal secretion) [9, 10]. In general, color-based presumptive tests (Phadebas, SALIgAE test) can be challenging to interpret for weak to trace positives or for mixtures with blood [11].

The main disadvantage of these biochemical tests is their destructive nature, resulting in sample loss for subsequent DNA analysis [12]. Since forensic samples are often found in low quantity, destruction of DNA should be avoided. Another disadvantage of the current biochemical tests is their specificity for only one biological matrix [1–3, 12–14]. This means that several cascade tests might be needed before the biologic nature of a certain sample or stain is uncovered. Also, when the identity of one biological matrix is determined, no or little effort is made to find out if the sample is a mixture of different biological matrices. This is due to the high cost of the tests, the fact that cascade testing is very time consuming and the increased loss of sample when multiple tests are performed [15].

Besides the identification of a certain biological matrix, determining the species of the donor of the sample can be equally important. For example, benzidine testing is used to determine the presence of blood, but to distinguish human blood from animal blood, other tests such as the hexagon-Obti test are needed [14, 16]. In addition, these tests can only indicate the presence or absence of blood from one specific species. If negative, another test has to be performed in order to potentially identify what animal species the blood originated from.

Therefore, forensic science calls for a universal, specific, and unbiased method that can identify pure as well as mixtures of biological matrices of different species origin without destruction of the DNA and preferably without any additional sample consumption, so that the full trace is available for DNA extraction.

Here, we present a mass spectrometry (MS)-based approach that can fulfill these requirements. In order to validate the use of mass spectrometry for the determination of biological matrices in forensic science, we first constructed a decision tree based on the specific and most prominent proteins present in biological matrices that might be found at a crime scene (blood, saliva, sperm, vaginal fluid, nasal secretion, feces, and urine). For the detection of semen, we focused on proteins present in the seminal fluid [4, 5, 12]. In this way, our approach could also be used to detect semen from men having undergone a vasectomy.

After the construction of a decision tree, the biomarkers for the most commonly encountered biological matrices in forensics (blood, semen, vaginal fluid, saliva) were evaluated in a blind analysis and all samples were annotated correctly, except when low amounts of saliva or vaginal fluid were present in combination with high amounts of blood or semen. Next, dilution series for saliva, blood, and semen proved our method equally or even more sensitive compared to other, biochemical methods. The MS approach could also detect semen on vaginal swabs 24 to 36 h after a possible rape. Additionally, species identification was validated on human, canine, and bovine blood. Finally, our decision tree was validated on real forensic samples. Constructing this methodology required a great amount of laboratory expertise, but new approaches in the field such as SRM (selected reaction monitoring) and library search algorithms are increasingly making MS-based detection methods accessible to a broader community. Therefore, our method could become a valuable addition to the forensic toolbox.

## Materials and methods

### Sample preparation

Different biological matrices were applied on sterile cotton-tipped swabs. The swabs were dried for 3 h at room temperature and stored at −20°C. For the validation of biomarkers, different biological matrices were applied, either alone or in mixtures (Table 1), on a piece of cotton clothing which was first rubbed on hands and arms to contaminate the fabric with human keratins. Additionally, the fabric was deliberately soiled with dirt on shoes and floor to mimic real life samples. The different spots were dried for 1 h.

**Table 1:** List of biological fluids and mixtures used in the blind experiment

Blind sample	Identifications	Identified biomarkers	Number of peptides	Protein score	Maximum *p* value
Z1	Semen	Semenogelin-1	39	1,094	1.1e−008
		Semenogelin-2	30	1,009	3.7e−012
		Prostatic acid phosphatase	4	156	1e−005
		Prostate-specific antigen	1	82	4.1e−006
Z2^a^	Vaginal secretion	Cornulin	1	81	5.2e−006
Z2 b^b^	Vaginal secretion	Cornulin	4	239	8.4e−008
		Cornifin	2	130	2.4e−008
Z3	Bovine blood	Hemoglobin subunit beta OS = *Bos taurus*	27	904	1.3e−007
		Hemoglobin subunit alpha OS = *Bos taurus*	15	847	4.5e−014
Z4	Human blood	Hemoglobin subunit beta	24	905	5.4e−010
		Hemoglobin subunit alpha	7	348	1.8e−007
Z5^a^	Saliva	Alpha-amylase 1	1	63	0.00034
Z5 b^b^	Saliva	Alpha-amylase 1	14	813	3.8e−011
Z6	Semen	Semenogelin-2	35	1,158	2.3e−009
		Semenogelin-1	32	828	6.9e−009
		Prostatic acid phosphatase	3	179	1.9e−007
		Prostate-specific antigen	2	113	1.5e−005
W	Semen, vaginal secretion	Semenogelin-2	21	737	1.3e−009
		Semenogelin-1	24	659	5.7e−006
		Cornulin	5	656	1.9e−022
		Prostatic acid phosphatase	1	89	8.3e−007
		Cornifin-B	2	84	0.00074
		Involucrin	1	46	0.018
X1	No biological matrix	/			
X2	Semen, human blood	Hemoglobin subunit beta	36	1,203	5.6e−010
		Hemoglobin subunit alpha	16	795	9.3e−017
		Semenogelin-2	6	271	2.2e−009
		Semenogelin-1	2	76	0.00016
X3	Human blood	Hemoglobin subunit beta OS = *Homo sapiens*	10	254	9.5e−008
		Hemoglobin subunit alpha OS = *Homo sapiens*	6	254	8.8e−008
X4	Semen, saliva	Semenogelin-2	35	1,793	8.1e−023
		Semenogelin-1	41	1,337	2.2e−015
		Alpha-amylase	6	244	6e−010
		Prostate-specific antigen	5	207	3.5e−006
		Prostatic acid phosphatase	5	205	1.7e−007
X5	Human blood, saliva	Hemoglobin subunit beta	39	1,187	5.9e−010
		Hemoglobin subunit alpha	15	807	2.6e−018
		Alpha-amylase 1	3	129	1.4e−005
X6	Bovine blood, saliva	Hemoglobin subunit beta OS = *Bos taurus*	24	860	1.3e−007
		Hemoglobin subunit alpha OS = *Bos taurus*	13	787	1.3e−015
		Alpha-amylase 1	3	160	3.1e−007
X7	Semen, vaginal secretion	Semenogelin-2	25	891	4.3e−010
		Semenogelin-1	28	712	9.4e−007
		Cornulin	2	219	1.9e−012
		Prostatic acid phosphatase	5	201	1e−007

The annotations were performed with Mascot Daemon. The number of identified peptides, the protein score, and the maximal expectancy are indicated as well

^a^1/10 of the sample gave a low number of peptides after MS analysis (n=1)

^b^1/2 of the sample was used for MS analysis

Before protein extraction, the swabs and the fabric were thawed, cut, and transferred into LoBind Eppendorf tubes (Eppendorf AG, Hamburg, Germany). After adding 500 μL of ultrapure water (MilliQ, Merck Millipore, Billerica, MA, USA), the Eppendorf tubes were vortexed thoroughly and the samples were incubated for 30 min at room temperature. Subsequently, the Eppendorf tubes were centrifuged for 5 min at 14,000×*g*. Since this procedure is the first step of the chelex DNA extraction, the pellet can be used for DNA analysis [17]. The supernatant is normally thrown away. Here, it is stored at −20°C and is later used for MS analysis.

The validation experiment was blind, meaning that the identity of the biological matrices in the different spots was not known to the investigator.

All volunteers who donated biological fluids consented to participation in the study.

### Sensitivity of the MS approach

Different dilutions of saliva (1:2, 1:3, 1:4, 1:5, 1:10, 1:100, 1:200, 1:500, 1:1,000; *n* = 3), human blood (1:10, 1:100, 1:1,000, 1:10,000, 1:100,000, 1:1,000,000; *n* = 3) and semen (1:10, 1:100, 1:1,000, 1:10,000, 1:100,000, 1:1,000,000; *n* = 3) were made in Eppendorf tubes with ultrapure water. Then 100 μL of the diluted saliva, 40 μL of the diluted blood, and 200 μL of the diluted semen were analyzed by means of the MS approach. The same volume of each dilution was used for the conventional biochemical methods. For saliva, 100 μL of the dilution was applied on cotton swabs and dried prior to the Phadebas test (Magle AB, Lund, Sweden). For blood, 40 μL of the dilution was applied on filter paper for the benzidine test. For semen detection, 200 μL of the dilution was directly applied on the Seratec PSA semiquant test (Seratec, Göttingen, Germany) for PSA detection, and for acidic phosphatase (AP) detection, 200 μL was applied on a piece of cotton fabric, dried, and analyzed with the Phosphatesmo Km Paper kit (Macherey-Nagel GmbH & Co., Düren, Germany). All the conventional tests were performed by trained experts according to the manufacturer’s protocols.

To validate the use of MS to find traces of semen on different time lapses after a rape, vaginal swabs were taken 12 h, 24 h, 36 h, 48 h, 60 h, and 72 h after sexual intercourse (*n* = 3). All swabs were analyzed as described above and compared with the Seratec PSA semiquant test for semen detection.

### Forensic samples

Real forensic samples (*n* = 12) were also analyzed to confirm the applicability of this technique in a real-world setting. Small pieces of fabric (0.5 cm²) were cut out from underwear or other clothing that was available from five different rape cases and divided in two: one part was tested for PSA or AP by means of conventional biochemical tests and the other part was used for mass spectrometric analysis. To this end, the pieces of clothing were incubated in 500 μL ultrapure water for 30 min as described above. One tenth of the sample was injected for mass spectrometric analysis. Additionally, seven other forensic samples that were positive for blood after a benzidine test were analyzed with MS.

### In solution digest

One twenty-fifth of the supernatant from the swabs with only one biological matrix was used to determine possible biomarkers, while 1/10 of the supernatant was used for the other samples (possible mixtures of biological matrices on the dirty fabric and forensic samples). Analysis of a higher amount of proteins for the samples from the dirty fabric and for the forensic samples is recommended since identification of biomarkers in these samples can be hampered by interfering proteins or dirt. For the blind samples with known proportions, a mixture of 100 μL was prepared and 1/100 of the sample was analyzed. Samples with a low number of identified peptides were rerun in a larger sample size [9/10 instead of 1/10 (Table 1) or 1/2 instead of 1/100 (Supplementary Table S2)]. Dried supernatant or dried body fluid mixtures were dissolved in 20 μL 0.5 M triethylammonium bicarbonate (TEABC; Sigma-Aldrich, St. Louis, MO, USA). One microliter of denaturant (2 % SDS; MP, Illkirch, France) and 2 μL reducing agent [50 mM tris-(2-carboxyethyl)phosphine (TCEP; Sigma-Aldrich, St. Louis, MO, USA)] were added to each Eppendorf and incubated for 1 h at 60°C. Subsequently, 1 μL of alkylizing agent [200 mM methyl methanethiosulfonate (MMTS; Sigma-Aldrich, St. Louis, MO, USA)] was added. After a 10-min incubation at room temperature, the proteins were digested overnight at 37°C with 1 μg of trypsin (Promega, Madison, WI, USA). The resulting peptides were dried and stored at −20°C.

### Mass spectrometric analysis

Dried peptides were dissolved in 40 μL 0.1 % formic acid (FA) in water (buffer A) and 20 μL was desalted after injection on a Acclaim PepMap 100 C18 pre-column [0.3 mm internal diameter (i.d.) × 5 mm, 5 μm particle size; Dionex, Sunnyvale, CA, USA] with buffer A. Separation was performed by means of reversed phase nano-HPLC (Pepmap C18 column 15 cm, particle size 3 μm, 0.3 mm internal diameter by 150 mm; Dionex, Sunnyvale, CA, USA) at 60°C using a linear gradient of 97:3 buffer A/buffer B to 20:80 buffer A/buffer B at 300 nL/min over 70 min (buffer B—80 % ACN/0.1 % FA). The different peptides were analyzed on an ESI Q-TOF Ultima (Waters, Milford, MA, USA) in a data dependent mode, where automatically switching between MS and MS/MS occurred on up to seven higher charge ions, when the intensity of the individual ions rose above 50 counts per second. The fluid was dispersed at a voltage between 1,800 V and 2,200 V (capillary voltage) and the cone voltage was set at 100. The source temperature was 95°C, while the dissolvation temperature was set at 120°C. *m*/*z* ratios were selected for MS between 450 and 1,650. MS/MS spectra were acquired between 50 and 2,300 Da. Ions were fragmented by collision induced dissociation, with a custom collision energy profile for LC–MSMS samples, ranging from 25 eV to 55 eV for doubly charged peptides between *m*/*z* 400 and 1,200, and ranging from 11 eV to 26 eV for triply charged peptides between *m*/*z* 435 and 1,000. *m*/*z* ratios selected for MS/MS were excluded for 150 s. Data were searched against Swissprot database of Mammalia using the in-house search engine Mascot Daemon (2.3; Matrix Science, London, UK). Methylthio (C) was specified as fixed modification since this modification was added to the peptides through alkylation by means of MMTS during the digest protocol. Oxidation (M) and deamidation (NQ) were considered as variable modifications since these are very common modifications on proteins/peptides [18–20].

**Fig. 1:**
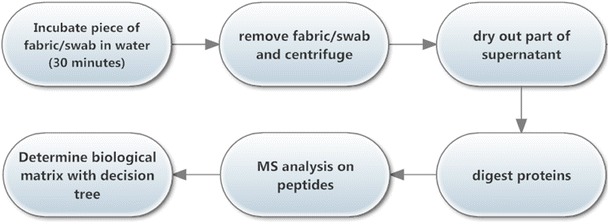
Workflow for mass spectrometric identification of biological matrices

The peptide tolerance and MS/MS tolerance were set to 0.35 Da and 0.6 Da, respectively. A maximum of two missed cleavages were allowed. To filter out homologous proteins, only the proteins with at least one bold red peptide in Mascot Daemon were used. Red indicates the top scoring peptide match for this spectrum and bold indicates that it is the highest scoring protein this peptide match is found in. By dropping hits that have no bold red matches, we can thus largely eliminate homologues with lower coverage [18]. In general, the identification threshold was set at a *p* value of 0.05 per peptide. The *p* value is the probability of a false positive annotation of a peptide. For the determination of the biomarkers, we decreased the *p* value to 0.01 to make sure that the identified proteins were not derived from false positive annotations of peptides. Searches were performed with trypsin as enzyme. For urine and feces, searches were performed with both trypsin and semitrypsin. The number of identified peptides is mentioned as a rough estimate of the abundance of this protein in the sample. The score of a peptide is a measure for the quality of the spectrum obtained after MSMS (threshold was set at 41) and the score of a protein is the sum of scores of all peptides annotated for that protein. Note that the *p* value can only be calculated for one peptide and not for the whole protein [18]. The *p* values in the tables are thus a measure for the false discovery rate of the best annotated peptide. The basic principles on proteomics and mass spectrometry are reviewed in [21, 22]. Automation of this approach will no longer require the interpretation of these scoring algorithms. The workflow of the mass spectrometric approach for the identification of biological matrices is depicted in Fig. 1.

## Results and discussion

### Biomarkers of fluid biological matrices

Nine biological matrices from different individuals [human blood (*n* = 5), animal blood (bovine and canine; *n* = 4), human menstrual blood (*n* = 3), semen (*n* = 2), vaginal fluid (*n* = 4), saliva (*n* = 2), nasal secretion (*n* = 5), feces (*n* = 4), and urine (*n* = 6)] were applied on sterile swabs in order to identify possible biomarkers. Supernatant of these samples were digested in peptides and used for mass spectrometric (MS) analysis. Per biological matrix different proteins were identified (*p* < 0.01) (Supplementary Data Table S1). Our selection criteria for biomarkers were based on the specificity and the abundance of the protein. Since the amount of biological matrix in forensic samples can be low, it has no use to incorporate biomarkers that can hardly be detected. Therefore, we chose our biomarkers from the highly abundant proteins.

For blood, hemoglobin (alpha and beta subunit) was chosen as biomarker since this protein was specific for blood and present in large amounts (Supplementary Data Table S1) [14, 16]. Mass spectrometry has already been used to identify hemoglobin variants in blood [23]. Additionally, hemoglobin is not only very specific and highly abundant in blood but it also allows to distinguish between different species of origin. Espinoza et al. found that hemoglobin, analyzed by electrospray ionization mass spectrometry, can resolve species by the presence of species determining peptides [24]. Despite extensive homology, all three species tested (*Homo sapiens*, *Canis familiaris*, and *Bos taurus*) were unambiguously annotated based on highly confident identification of species-specific peptide stretches. The hexagon Obti test that is most commonly used to determine human origin of blood stains can differentiate between human and non-human blood, but MS can easily pinpoint the species as well, again without any loss of trace or cellular material needed for subsequent DNA analysis.

**Fig. 2:**
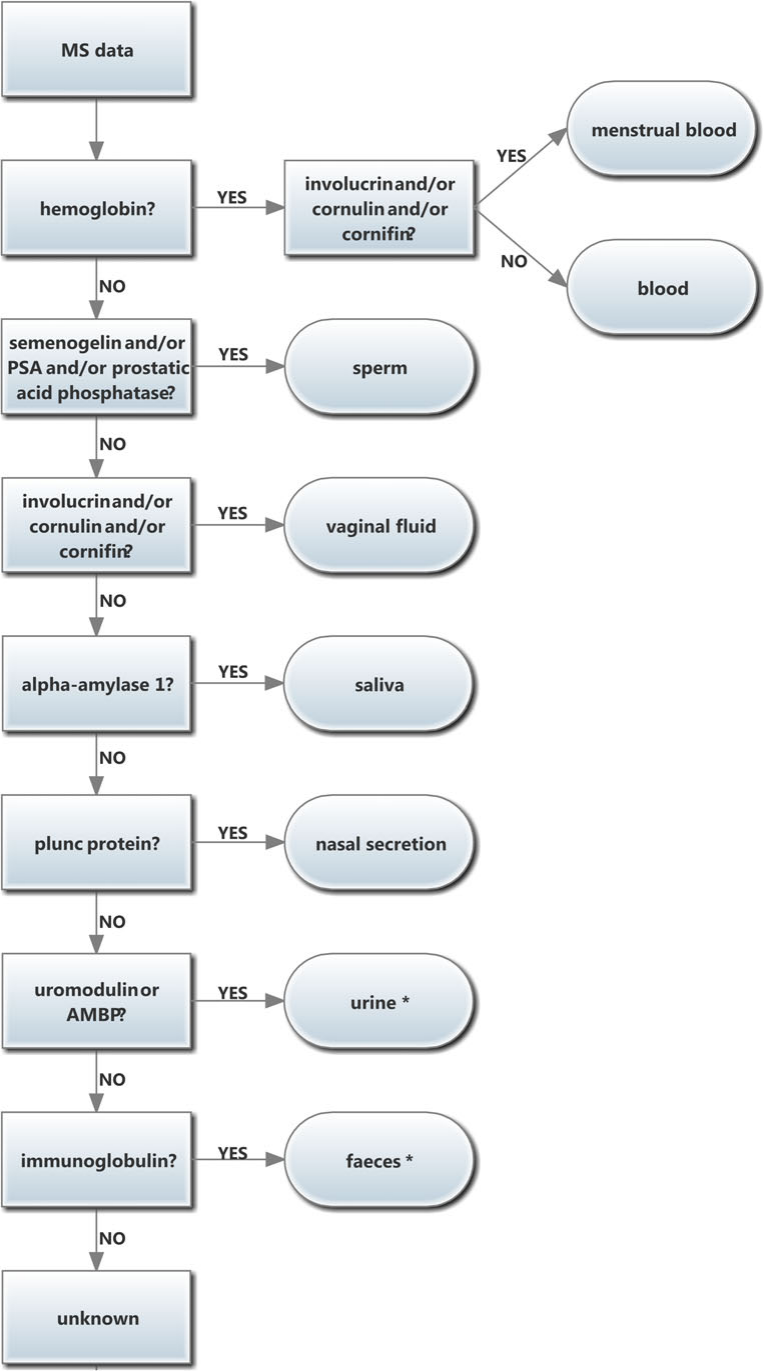
Decision tree with biomarkers per biological matrix. *The absence of the biomarker (uromodulin/AMBP protein or immunoglobulins) does not necessarily exclude the presence of the matrix (urine or feces, respectively)

By means of mass spectrometry, semenogelin 1 and 2 but also other semen-specific proteins such as prostatic acid phosphatase and PSA were found (Fig. 2). By means of the MS approach, these different proteins are often detected in one single test and in this way the presence of different semen proteins further confirms the origin of the matrix.

Vaginal biomarkers are cornulin, involucrin, and cornifin while alpha-amylase 1 is the highly abundant and specific protein for saliva. By means of mass spectrometry, a distinction between the two forms of alpha-amylase (alpha-amylase 1, present in saliva and alpha-amylase 2, present in semen and vaginal secretion) can be made, thus increasing specificity.

In menstrual blood, the biomarkers from both blood and vaginal secretions were present: hemoglobin and cornulin.

For nasal secretions, albumin, immunoglobulins (Ig alpha-chain, Ig kappa chain C region, J chain) and plunc protein were detected. The latter was specific for this matrix [25, 26].

In urine, a few proteins/peptides could be detected: albumin, AMBP protein, and uromodulin. These proteins have already been detected previously [27–29]. Albumin is found in high amounts in blood as well and can therefore not be used as biomarker. Uromodulin, on the other hand, is the most abundant protein in normal human urine [30]. It should be noted that it can also be detected in serum [31]. However, when no hemoglobin is found in a sample, we can exclude blood as biological fluid. In this way, uromodulin can serve as a biomarker for urine in the absence of the manifold more abundant hemoglobin that would show up when blood is present. Similarly, the presence of AMBP protein in the absence of hemoglobin can be used as a marker for urine. However, the absence of uromodulin or AMBP protein does not mean that no urine is present since in some urine samples no proteins or peptides could be detected, probably due to their low concentration.

For feces, immunoglobulins (J chain, Ig kappa chain C region, and Ig alpha chain) could be detected in some samples (two out four samples). However, immunoglobulins are not ideal biomarkers for the identification of biological matrices since these proteins are also found in other matrices such as blood. Still, the presence of immunoglobulins and/or albumin in the absence of hemoglobin can indicate the presence of feces and excludes the presence of blood. Therefore, a feces trace will not be mistaken for a blood trace, but mixtures of blood and feces will be hard to identify as a mixture. A decision tree with biomarkers for different biological matrices is depicted in Fig. 2.

### Blind testing and annotation of fluid biological matrices

In order to evaluate our list of biomarkers, the most relevant biological matrices in forensic casework (human blood, non-human blood, semen, saliva, and vaginal secretions) were applied on a dirty cotton fabric in order to mimic real-life situations. Since the biomarkers for urine and feces could not always be detected and since urine, feces, and nasal secretions are seldom crucial in forensic casework, we did not incorporate them in further analyses.

Mixtures of several biological matrices were also included as this often occurs in real casework (Table 1). A major advantage of this unbiased MS approach is the ability to annotate different proteins at the same time, making it possible to identify several biological matrices in one sample with only one test. This equally makes cascade testing redundant. Additionally, different markers for the same matrix such as semenogelin and PSA for semen can be used as a confirmation of the identity of that matrix.

During this analysis, no information on the presence of biological fluids or the presence of mixtures was available to the investigator. Samples Z2 and Z5 showed a relatively low number of peptides. Therefore, these samples were rerun with 1/2 of the sample instead of 1/10. All identifications were correct, which clearly demonstrates the robustness and specificity of our decision tree.

Additionally, we tested these matrices in different proportions and applied 1/100 of the sample on the mass spectrometer (Supplementary Data Table S2). Moreover, 1/100 of the sample was used since the amount of biological fluid to make these samples was much higher than the amount present in forensic samples (see “Materials and methods”). It should be noted, however, that one sample (S3) showed a low number of peptides. Therefore, 1/2 instead of 1/100 of this sample was rerun on the mass spectrometer. All four matrices were correctly annotated, including low amounts of blood and semen in high amounts of another biological matrix. However, low amounts of saliva and vaginal fluid, two matrices with low protein concentrations, could not be detected when mixed in high amounts of blood and semen, the matrices with high protein concentrations.

All identifications with a score above 41, which is correlated with a *p* value below 0.05, are mentioned in the table (Table S2). It is worth mentioning that in sample S5, prostatic acid phosphatase (one peptide, score 38, maximum *p* value of 0.071) could also be detected. This peptide had a score below the threshold, but it can be seen as a confirmation of semen by means of semenogelin detection. Similarly, prostatic acid phosphatase (two peptides, score 32, maximum *p* value 0.39) and prostate-specific antigen (one peptide, score 35, maximum *p* value 0.22) were also found in sample S10.

### Sensitivity of the mass spectrometric approach

In order to determine the sensitivity of the mass spectrometric approach, we made dilutions of the biological fluids of interest. For semen, saliva, and blood, these sensitivities were compared with the conventional biochemical methods (Seratec PSA semiquant test for PSA and the Phosphatesmo Km Paper kit for AP were used for semen, benzidine for the detection of blood, and the Phadebas test for saliva) for samples from three different volunteers per biological matrix. The MS approach showed comparable or even higher sensitivity when compared to the currently used biochemical tests. The maximum sensitivity of the benzidine test was 1:10,000 in our laboratory, while mass spectrometry showed a sensitivity of 1:100,000 starting from the same amount of sample. However, it should be noted that luminol has also been reported with a sensitivity of 1:100,000 [1]. For saliva, a maximum sensitivity of 1:100 for the Phadebas test was observed in our laboratory. The MS approach reveals a maximum sensitivity of 1:1,000. For semen, a higher specificity compared to the AP test (1:100) and a comparable sensitivity compared to the PSA test (1:100,000) was found by means of MS (maximum sensitivity of 1:100,000). Taken together, the sensitivity of the mass spectrometric approach is comparable to the conventional methods or even exceeds their performance.

Since forensic science is often used to solve rape cases, a time-lapse experiment was also conducted to determine the sensitivity of post-coital sperm detection. After sexual intercourse, a vaginal swab was obtained from three volunteers every 12 h. By means of MS, the presence of semen (semenogelin) could still be detected after 24 h (*n* = 3) or even after 36 h (*n* = 2). When using the same amount of sample, the Seratec PSA semiquant test could only detect semen up to 12 h after intercourse in all three volunteers.

### Annotation of forensic samples

As a final validation of our approach, we analyzed real forensic samples from actual cases. Five different rape cases with semen on clothing were analyzed with the conventional methods to detect semen. For MS analysis, only the supernatant was analyzed while the pelleted cells were used for DNA profiling. Not only in recent samples (2010 and 2011) but also in 5–8-year-old samples (a sample of 2006 and a sample of 2003) semen and/or vaginal fluid could be detected by means of the MS approach. A forensic sample with semen on a sweater revealed the presence of semen (semenogelin, PSA) and the absence of vaginal fluid (no involucrin, cornulin, or cornifin) (Table 2). Interestingly, alpha-amylase 1 was also detected, which revealed the presence of saliva. The latter was not determined by the conventional method because after identification of one biological matrix (such as semen in a rape case), testing is usually abrogated.

**Table 2:** Detection of biological matrices on forensic samples from rape cases, with their number of identified peptides, the score, and maximum *p* value from Mascot Daemon

Sample (age)	Identified biomarkers	Identified matrix	Number of peptides	Protein score	Maximum *p* value
Washcloth (6 months)	Semenogelin-1	Sperm	4	174	1e−005
Women’s sweater (1 year)	Semenogelin-2	Sperm	19	819	4.6e−017
	Semenogelin-1		15	630	2.7e−015
	Prostatic acid phosphatase		4	125	0.00029
	Alpha-amylase 1	Saliva	2	197	2.8e−015
Women’s underpants (2 years)	Prostatic acid phosphatase	Sperm	1	73	3.5e−005
	Cornifin-A	Vaginal fluid	3	44	0.03
Women’s underpants (5 years)	Semenogelin-2	Sperm	18	510	2.9e−008
	Semenogelin-1		20	492	4.3e−008
	Prostatic acid phosphatase		5	152	4e−005
	Prostate-specific antigen		1	72	4.9e−005
	Cornulin	Vaginal fluid	1	91	5.3e−007
Women’s underpants (8 years)	Prostate-specific antigen	Sperm	4	182	3.2e−007
	Cornulin	Vaginal fluid	1	67	0.00015

Besides rape cases, real forensic samples with possible blood stains were included as well. Supernatants from stains (max 1 year old) that were positive for blood with the conventional test (benzidine) were analyzed by means of mass spectrometry. In each sample, hemoglobin was detected (Table 3), again illustrating the usefulness of mass spectrometry for identification of the biological fluids in forensic caseworks.

**Table 3:** Detection of blood on forensic samples by means of mass spectrometry

Sample	Identified biomarkers	Number of peptides	Protein score	Maximum *p* value
Swab from car door	Hemoglobin subunit beta	36	968	1.3e−012
	Hemoglobin subunit alpha	25	818	6.1e−011
Swab from car door	Hemoglobin subunit beta	6	140	3.4e−006
	Hemoglobin subunit alpha	3	53	0.037
Swab from stain on back of envelope	Hemoglobin subunit beta	7	127	1.6e−005
	Hemoglobin subunit alpha	4	93	7.6e−007
Swab, origin unknown	Hemoglobin subunit beta	32	905	3e−013
	Hemoglobin subunit alpha	18	584	7.1e−013
Swab from a sweater	Hemoglobin subunit beta	16	555	1.2e−013
	Hemoglobin subunit alpha	13	358	2.6e−008
Swab from stain on envelope	Hemoglobin subunit beta	43	838	2.4e−010
	Hemoglobin subunit alpha	16	354	7.2e−007
Swab from shower	Hemoglobin subunit beta	39	768	4.9e−010
	Hemoglobin subunit alpha	23	479	2.6e−011

The number of identified peptides, the score, and the maximum *p* value from Mascot Daemon are indicated as well

We also tested the potential of the approach to discriminate animal traces in real forensic samples. In the stomach of a corpse, brains were found, but no information was known on the species identity. MS analysis revealed the presence of canine hemoglobin (two peptides, maximum *p* value = 0.0003, score = 64), indicating that the brains originated from dogs. In another case, blood was found on the floor at a crime scene, but no DNA profile could be obtained. By means of this MS approach, we found that the blood originated from a dog (11 peptides from hemoglobin beta, origin *C. familiaris*, score of 288, maximum *p* value of 6.6e−006; four peptides from hemoglobin alpha, origin *C. familiaris*, score of 181, maximum *p* value of 3.2e−010).

### Biological matrix identification in practice

To further optimize this approach for implementation, automated digestion can be used to diminish the workload: shorter digestion times have already been described (2.5 s–7.5 min) and will dramatically decrease the sample preparation time [32, 33]. Data analysis can be simplified by developing a selected reaction monitoring (SRM) workflow on proteotypic peptides [34]. Importantly, the use of MS analysis in forensics has the additional advantage that every peptide identification is linked with a *p* value. Thus, the certainty about the identity of a sample can be translated into a probability, while interpretation of biochemical tests can be subjective when low amounts of sample are present. This *p* value can more easily be used in court, as is currently done for presenting DNA typing results. It should be noted, however, that the algorithm calculates these probabilities for each separate MSMS spectrum (peptide) as independent *p* values and that these cannot simply be transposed to a probability of the protein identification as a whole. This means that the easiest way to present statistical data is by presenting the best peptide hit if the *p* value is very low. On the other hand, a protein that is identified by three different peptide stretches of each *p* = 0.05 should be seen as a very strong indication of the presence of the protein. This expert data handling, however, is expected to be overcome following future automation steps.

Notably, this approach was optimized and validated in parallel with chelex extraction for DNA typing. Other DNA extraction kits, based on silica for example, were not tested, but since our approach is performed on the extracellular proteins, which can easily be isolated in advance, the DNA extraction procedure has no influence on the MS results.

The use of mass spectrometry in forensic science has been reported before, mainly in toxicology studies, such as the detection of opioids or ephedrines [35–37]. But mass spectrometry can also differentiate between different sources of the same material based on their isotope ratio [38] and can be used for genotyping and the determination of single nucleotide polymorphisms [39–41]. However, to the authors’ knowledge, this is the first study that highlights the use of mass spectrometry to identify biological matrices in forensic science.

### Mapping the MS approach in current forensic sciences

Besides mass spectrometry on proteins, other techniques have been suggested to identify the matrix and the species it is derived from. Techniques focusing specifically on mRNA, miRNA, and DNA methylation, but also more general detection methods such as fluorescence spectroscopy and RAMAN spectroscopy, have proven useful in the field. Species identification by means of mRNA markers relies on the unique expression of mRNA to identify body fluid stains [15, 42, 43]. However, cross-reactivity is still an issue with this technique and novel mRNA markers for all body fluids are needed to increase the discriminatory power of the assay. To the author’s knowledge, no clear consensus on the choice of mRNA markers has currently been achieved [42]. Additionally, the stability of mRNA can lead to problems in the forensic context [44, 45]. Therefore, a lot of attention is currently paid to microRNA (miRNA) as a tool to identify biological matrices. Because of their small size, they are less prone to degradation, unlike the larger mRNAs [46, 47]. Hanson et al. were the first to use miRNA profiling as an alternative approach to body fluid identification in forensic casework. They found a panel of nine miRNA that allowed them to differentiate between blood, semen, saliva, vaginal secretions, and menstrual blood. However, no strong miRNA candidate was found for semen. It should be noted that a more recent study by Zubakov et al. could not reproduce these results [47]. Possible explanations are the difference in strategy for both cDNA synthesis and PCR quantification, and also the natural variation in miRNA expression between individuals. So, rigorous methodological validation and standardization are crucial.

miRNA can be used to test different biological matrices in one sample, but the detection of each miRNA must be performed in different wells and thereby in different analyses. Multiplexing is possible but is limited due to the limited number of dyes available for use in QT-PCR assays [48]. By means of the MS approach, no multiplexing is necessary since all biological matrices can be analyzed in one single run.

Recently, Frumkin et al. reported a new method to identify forensic tissue based on methylation of DNA [49, 50]. This technique based on tissue-specific methylation patterns has a lot of advantages since it gives operator-independent results, similar to the MS approach, and can be multiplexed with existing STR typing protocols without additional sample loss. Importantly, this technique is still cell-based, which makes it unsuited for detecting sperm from men with a vasectomy and implying a need for more DNA for multiplexing (1 ng versus 100 pg necessary for a good DNA profile) [51]. Frumkin et al. also rightfully state that the loss of function of proteins can lead to false negatives in the current protein-based commercial kits [49]. However, the primary structure of proteins is extremely stable and its sequence can still be identified by means of mass spectrometry after hundreds of years [52], which makes them perfectly suited for forensic science.

Fluorescence spectroscopy can also be used for forensic casework, but despite its good sensitivity, questions arise about its specificity because the fluorescence emission peaks are very broad [12]. This also makes it harder to identify possible mixtures of biological fluids. Finally, RAMAN spectroscopy, based on scattering of light, has a higher specificity since very narrow peaks are formed. However, its sensitivity is lower. RAMAN can identify mixtures and is able to distinguish human from canine semen [53], but no difference between cat, dog, and human blood was found in that study [14]. By means of mass spectrometry, on the other hand, high sensitivity and specificity can be obtained and species identification can be performed at the same time.

Our MS method has therefore a lot of advantages, but it should be noted as well that at this moment it is not suited to replace the current (colorimetric, enzymatic,…) methods to identify biological matrices in forensic science. Although the protocol for this method is easy to perform, it takes longer before obtaining results compared to the current biochemical tests. Despite the fact that portable mass spectrometers are already available, tissue identification at a crime scene is not feasible at the moment. However, there is no doubt that this technique is useful as an additional method and that it can be adapted in the future for easier application.

In conclusion, by means of this MS-based proteomics approach, we were able to distinguish all the frequently presented samples in forensic casework (vaginal fluid, semen, saliva, and blood) as well as mixtures thereof and could easily annotate animal contamination. The major advantage of this technique is the fact that the analysis is not destructive for DNA since it is performed on the supernatant of the sample, which is otherwise discarded, so the pellet can still be used for DNA typing. Therefore, this technique can be used either as an exploration or as a confirmation technique, when questions arise upon the identity of the biological matrix after DNA profiling in combination with traditional screening tests. Additionally, by means of MS, species annotation and identification of the biological matrix can be performed in one single test instead of cascade testing. This is of major importance as more often than not these traces are scarce on a crime scene. Finally, the high sensitivity of this approach and the stability of proteins make this method fitted to identify both small and old samples.

## Electronic supplementary material

Below is the link to the electronic supplementary material.

ESM 1 (DOCX 48 kb)
