# Supplementary material for: Mass spectrometry-based proteomics as a tool to identify biological matrices in forensic science
Source: Int J Legal Med. 2012 Jul 29;127(2):287–98. doi: 10.1007/s00414-012-0747-x (PMC3578717; doi:10.1007/s00414-012-0747-x)
Supplement: Supplementary file 1 — (DOCX 48 kb) [file 414_2012_747_MOESM1_ESM.docx]

**Submission to International Journal of Legal Medicine:**

**MS-based Proteomics as a tool to identify biological matrices in forensic research**

**Katleen Van Steendam (PhD, PharmD), Marlies De Ceuleneer (PharmD), Maarten Dhaenens (PhD), David Van Hoofstat (PhD), Dieter Deforce (PhD, PharmD).**

**Laboratory for Pharmaceutical Biotechnology, Ghent University, Harelbekestraat 72, B-9000 Ghent, Belgium**

Corresponding author: Dieter Deforce

Harelbekestraat 72

9000 Gent

Belgium

Phone: 0032 9 264 80 67

Fax: 0032 9 220 66 88

Email: dieter.deforce@ugent.be

**Supplementary data**

Table S1

**Table S1: annotated proteins in different biological matrices (threshold was set at p<0.01 and at a score of 41).**

If a protein was annotated in several species, only the species with the highest score/p-value was mentioned in the table since the presence of several species is due to homology.

| Matrix | Identification | Species | Protein score | Maximum *p* value |
| --- | --- | --- | --- | --- |
| Urine | Serum albumine | Homo sapiens | 81 | 1.9e-006 |
|  | Uromoduline | Homo sapiens | 66 | 0.00018 |
|  |  |  |  |  |
| Faeces | Immunoglobulin J chain | Homo sapiens | 80 | 6.6e-006 |
|  | Ig kappa chain C region | Homo sapiens | 74 | 2.8e-005 |
|  | Ig alpha-2 chain C region | Homo sapiens | 72 | 0.002 |
|  |  |  |  |  |
| Nasal secretion | Serum albumin | Homo sapiens | 391 | 2.8e-009 |
|  | Ig alpha-2 chain C region | Homo sapiens | 237 | 3.6e-009 |
|  | Ig alpha-1 chain C region | Gorilla gorilla gorilla | 161 | 2.9e-005 |
|  | Immunoglobulin J chain | Homo sapiens | 140 | 5.8e-008 |
|  | Ig kappa chain C region | Homo sapiens | 140 | 4.6e-009 |
|  | Lactotransferrin | Homo sapiens | 116 | 1.3e-007 |
|  | Zinc-alpha-2-glycoprotein | Homo sapiens | 97 | 1.4e-007 |
|  | Deleted in malignant brain tumors 1 protein | Homo sapiens | 95 | 2.3e-007 |
|  | Ig kappa chain V-I region DEE | Homo sapiens | 85 | 2.1e-006 |
|  | Ig lambda-1 chain C regions | Homo sapiens | 74 | 3.2e-005 |
|  | Ig kappa chain V-II region 26-10 | Homo sapiens | 72 | 4.2e-005 |
|  | Protein S100-A7A | Homo sapiens | 70 | 6.4e-005 |
|  | Polymeric immunoglobulin receptor | Homo sapiens | 54 | 0.0031 |
|  | Prolactin-inducible protein homolog | Homo sapiens | 51 | 0.0051 |
|  | Protein Plunc | Homo sapiens | 51 | 0.0062 |
|  |  |  |  |  |
| Vaginal fluid | Cornulin | Homo sapiens | 677 | 1.00e-22 |
|  | Keratin. type I cytoskeletal 13 | Homo sapiens | 238 | 6.7e-009 |
|  | Keratin. type II cytoskeletal 6A | Homo sapiens | 195 | 1.9e-007 |
|  | Small proline-rich protein 3 | Homo sapiens | 191 | 2.5e-008 |
|  | Ig kappa chain C region | Homo sapiens | 170 | 1.5e-010 |
|  | Keratin. type II cytoskeletal 4 | Homo sapiens | 100 | 1.8e-005 |
|  | Ig gamma-1 chain C region | Homo sapiens | 94 | 2.4e-007 |
|  | Involucrin | Homo sapiens | 93 | 4.3e-006 |
|  | Cornifin-B | Homo sapiens | 90 | 0.00024 |
|  | Filaggrin | Homo sapiens | 86 | 2.00e-06 |
|  | Ig gamma-2 chain C region | Homo sapiens | 80 | 6.5e-006 |
|  | Serum albumin | Homo sapiens | 68 | 0.002 |
|  | Ig lambda-1 chain C regions | Homo sapiens | 65 | 0.00024 |
|  | Annexin A2 | Homo sapiens | 60 | 0.00064 |
|  | Thioredoxin | Homo sapiens | 58 | 0.0012 |
|  | Protein S100-A8 | Homo sapiens | 56 | 0.0018 |
|  | Serpin B3 | Homo sapiens | 54 | 0.003 |
|  |  |  |  |  |
| Saliva | Alpha-amylase 1 | Homo sapiens | 456 | 4.5e-010 |
|  | Serum albumin | Homo sapiens | 125 | 3.8e-006 |
|  | Ig alpha-1 chain C region | Homo sapiens | 84 | 7.5e-005 |
|  | Ig alpha-2 chain C region | Homo sapiens | 75 | 0.00066 |
|  | Ig kappa chain C region | Homo sapiens | 63 | 0.00042 |
|  | Submaxillary gland androgen- | Homo sapiens | 51 | 0.0062 |
|  | regulated protein 3B |  |  |  |
|  |  |  |  |  |
| Sterile sperm | Semenogelin-2 | Homo sapiens | 1726 | 1.2e-008 |
|  | Semenogelin-1 | Homo sapiens | 1236 | 8.8e-009 |
|  | Cystatin-SN | Homo sapiens | 104 | 3.2e-008 |
|  | Prostatic acid phosphatase | Homo sapiens | 94 | 3.2e-007 |
|  | Lactotransferrin | Homo sapiens | 89 | 0.00025 |
|  | Alpha-2-antiplasmin | Homo sapiens | 84 | 2.7e-006 |
|  | Serum albumin | Homo sapiens | 72 | 4.1e-005 |
|  | Clusterin | Homo sapiens | 72 | 0.002 |
|  | Cystatin-C | Homo sapiens | 63 | 0.00037 |
|  | Ellis-van Creveld syndrome protein homolog | Homo sapiens | 51 | 0.0054 |
|  |  |  |  |  |
| Sperm | Semenogelin-2 | Homo sapiens | 1841 | 4.8e-009 |
|  | Semenogelin-1 | Homo sapiens | 1714 | 1.6e-009 |
|  | Clusterin | Homo sapiens | 202 | 2.8e-008 |
|  | Prostatic acid phosphatase | Homo sapiens | 195 | 1.6e-008 |
|  | Prolactin-inducible protein | Homo sapiens | 175 | 3.1e-005 |
|  | Lactotransferrin | Homo sapiens | 165 | 0.0002 |
|  | Serum albumin | Homo sapiens | 143 | 3.3e-005 |
|  | Mucin-6 | Homo sapiens | 124 | 4.7e-006 |
|  | WAP four-disulfide core domain protein 2 | Homo sapiens | 95 | 1.2e-005 |
|  | Prostate-specific antigen | Homo sapiens | 91 | 0.00025 |
|  | Alpha-2-antiplasmin | Homo sapiens | 76 | 2.00e-05 |
|  | Proactivator polypeptide | Homo sapiens | 57 | 0.0015 |
|  | Cathepsin D | Canis familiaris | 56 | 0.0019 |
| Menstrual blood | Hemoglobin subunit beta | Homo sapiens | 944 | 4.5e-007 |
|  | Serum albumin | Homo sapiens | 750 | 1.1e-008 |
|  | Hemoglobin subunit alpha | Homo sapiens | 606 | 1.4e-010 |
|  | Hemoglobin subunit delta | Homo sapiens | 520 | 1.3e-005 |
|  | Annexin A1 | Homo sapiens | 416 | 3.1e-008 |
|  | Ig gamma-2 chain C region | Homo sapiens | 188 | 0.00018 |
|  | Cornulin | Homo sapiens | 188 | 7.9e-010 |
|  | Ig kappa chain V-II region 26-10 | Homo sapiens | 113 | 3.9e-009 |
|  | Ig kappa chain C region | Homo sapiens | 110 | 1.1e-005 |
|  | Small proline-rich protein 3 | Homo sapiens | 95 | 0.00027 |
|  | Annexin A2 | Homo sapiens | 89 | 5.2e-005 |
|  | Alpha-2-macroglobulin | Homo sapiens | 87 | 0.00015 |
|  | Ig alpha-1 chain C region | Homo sapiens | 84 | 0.00032 |
|  | Catalase | Homo sapiens | 81 | 5.2e-006 |
|  | Ig kappa chain V-I region CAR | Homo sapiens | 80 | 7.6e-006 |
|  | Thioredoxin | Homo sapiens | 64 | 0.00033 |
|  | Haptoglobin | Homo sapiens | 56 | 0.0039 |
|  | Fibrinogen gamma chain | Homo sapiens | 55 | 0.0025 |
|  | Serotransferrin | Homo sapiens | 54 | 0.0028 |
|  | Neuroblast differentiation-associated | Homo sapiens | 53 | 0.004 |
|  | protein AHNAK |  |  |  |
|  |  |  |  |  |
| Bovine blood | Serum albumin | Bos taurus | 1972 | 9.1e-016 |
|  | Hemoglobin subunit beta | Bos taurus | 1799 | 9.00e-15 |
|  | Hemoglobin subunit alpha | Bos taurus | 1663 | 3.3e-017 |
|  | Fibrinogen gamma-B chain | Bos taurus | 528 | 7.2e-020 |
|  | Alpha-2-macroglobulin | Bos taurus | 412 | 1.3e-016 |
|  | Fibrinogen beta chain | Bos taurus | 409 | 2.6e-012 |
|  | Peroxiredoxin-2 | Bos taurus | 317 | 4.00e-14 |
|  | Hemopexin | Bos taurus | 262 | 8.2e-013 |
|  | Alpha-2-HS-glycoprotein | Bos taurus | 240 | 2.4e-012 |
|  | Carbonic anhydrase 2 | Bos taurus | 221 | 1.2e-012 |
|  | Apolipoprotein A-I | Bos taurus | 214 | 8.2e-011 |
|  | Inter-alpha-trypsin inhibitor H4 | Bos taurus | 181 | 5.1e-016 |
|  | Fibrinogen alpha chain | Bos taurus | 171 | 7.00e-09 |
|  | Serotransferrin | Bos taurus | 129 | 3.9e-007 |
|  | Serpin A3-1 | Bos taurus | 129 | 6.7e-007 |
|  | Alpha-1B-glycoprotein | Bos taurus | 128 | 3.6e-006 |
|  | Apolipoprotein C-III | Bos taurus | 116 | 3.2e-008 |
|  | Vitamin D-binding protein | Bos taurus | 111 | 2.6e-006 |
|  | Alpha-1-antiproteinase | Bos taurus | 110 | 6.8e-009 |
|  | Complement C3 | Bos taurus | 75 | 2.00e-05 |
|  | Apolipoprotein A-II | Bos taurus | 63 | 0.00032 |
|  | Protein HP-25 homolog 2 | Bos taurus | 59 | 0.001 |
|  | Kininogen-1 | Bos taurus | 56 | 0.002 |
|  | Actin. cytoplasmic 1 | Bos taurus | 54 | 0.0032 |
|  |  |  |  |  |
| Human blood | Hemoglobin subunit beta | Homo sapiens | 2743 | 4.1e-011 |
|  | Serum albumin | Homo sapiens | 2557 | 4.1e-018 |
|  | Hemoglobin subunit alpha | Homo sapiens | 2378 | 1.8e-019 |
|  | Hemoglobin subunit delta | Homo sapiens | 1838 | 1.2e-010 |
|  | Carbonic anhydrase 1 | Homo sapiens | 463 | 3.7e-013 |
|  | Ig gamma-1 chain C region | Homo sapiens | 379 | 1.3e-013 |
|  | Ig gamma-4 chain C region | Homo sapiens | 296 | 6.8e-013 |
|  | Ig kappa chain C region | Homo sapiens | 290 | 9.1e-011 |
|  | Peroxiredoxin-2 | Homo sapiens | 214 | 9.4e-014 |
|  | Apolipoprotein A-I | Homo sapiens | 171 | 5.2e-007 |
|  | Serotransferrin | Homo sapiens | 167 | 7.7e-009 |
|  | Ig gamma-2 chain C region | Homo sapiens | 166 | 4.4e-009 |
|  | Apolipoprotein A-II | Homo sapiens | 161 | 7.00e-12 |
|  | Ig alpha-1 chain C region | Homo sapiens | 156 | 2e-010 |
|  | Fibrinogen alpha chain | Homo sapiens | 147 | 1.1e-008 |
|  | Alpha-1-antitrypsin | Homo sapiens | 125 | 2.7e-007 |
|  | Ig lambda-1 chain C regions | Homo sapiens | 122 | 3.9e-008 |
|  | Alpha-2-macroglobulin | Homo sapiens | 103 | 1.2e-005 |
|  | Fibrinogen gamma chain | Homo sapiens | 92 | 3e-007 |
|  | Catalase | Homo sapiens | 72 | 4.2e-005 |
|  | Carbonic anhydrase 2 | Homo sapiens | 67 | 0.00014 |
|  | Chaperone activity of bcl complex-like, mitochondrial | Bos taurus | 48 | 0.0097 |
| Dog blood | Hemoglobin subunit alpha | Canis latrans | 864 | 4.9e-021 |
|  | Hemoglobin subunit alpha | Canis familiaris | 840 | 0.00053 |
|  | Hemoglobin subunit beta | Canis familiaris | 661 | 1.6e-009 |
|  | Serum albumin | Canis familiaris | 184 | 3.3e-006 |
|  | Apolipoprotein A-I | Canis familiaris | 146 | 1.1e-005 |
|  | Peroxiredoxin-2 | Homo sapiens | 83 | 1.5e-006 |
|  | Carbonic anhydrase 2 | Homo sapiens | 70 | 1.00e-05 |
|  | Haptoglobin | Canis familiaris | 51 | 0.0037 |

Supplementary table 2

# Table S2: List of mixtures of biological fluids in known proportions in a blind experiment. The annotations were performed with Mascot Daemon. The number of identified peptides, the protein score and the maximal expectancy are indicated as well.

* 1/100 of the sample gave a low number of peptides after MS analysis (n=1)

** 1/2 of the sample was used for MS analysis

| Blind sample | Identifications | Identified biomarkers | Number of peptides | Protein score | Maximum *p* value |
| --- | --- | --- | --- | --- | --- |
| S1 | Saliva (90%) | Alpha-amylase 1 | 4 | 116 | 9.4e-006 |
|  | Semen (10%) | Semenogelin-2 | 1 | 68 | 0.00013 |
| S2 | Blood (90%) | Hemoglobin subunit beta OS=Homo sapiens | 38 | 1655 | 1.7e-014 |
|  |  | Hemoglobin subunit alpha OS=Homo sapiens | 26 | 1224 | 5.5e-014 |
|  | Saliva (10%) | / | / | / | / |
| S3* | Vaginal fluid (90%) | Cornulin | 1 | 43 | 0.034 |
|  | Semen (10%) | / | / | / | / |
| S3b ** | Vaginal fluid (90%) | Cornulin | 3 | 213 | 1.5e-011 |
|  |  | Cornifin-B | 3 | 133 | 2.5e-008 |
|  |  | Involucrin | 3 | 128 | 3.5e-007 |
|  |  | Cornifin-A | 2 | 116 | 4.1e-008 |
|  | Semen (10%) | Semenogelin-2 | 11 | 438 | 9.2e-008 |
|  |  | Semenogelin-1 | 3 | 242 | 5.6e-007 |
| S4 | Semen (90%) | Semenogelin-2 | 5 | 216 | 2.6e-006 |
|  |  | Semenogelin-1 | 4 | 123 | 0.00098 |
|  |  | Prostate-specific antigen | 1 | 65 | 0.00022 |
|  | Saliva (10%) | / | / | / | / |
| S5 | Blood (90%) | Hemoglobin subunit alpha OS=Homo sapiens | 23 | 1647 | 3.7e-015 |
|  |  | Hemoglobin subunit beta OS=Homo sapiens | 30 | 1152 | 1.2e-010 |
|  | Semen (10%) | Semenogelin-1 | 1 | 71 | 4.7e-005 |
|  |  | Semenogelin-2 | 1 | 56 | 0.00049 |
|  |  |  |  |  |  |
| S6 | Blood (90%) | Hemoglobin subunit beta OS=Homo sapiens | 36 | 1354 | 1.8e-012 |
|  |  | Hemoglobin subunit alpha OS=Homo sapiens | 24 | 849 | 8.9e-011 |
|  | Vaginal fluid (10%) | / | / | / | / |
| S7 | Saliva (90%) | Alpha-amylase 1 | 3 | 129 | 7.6e-006 |
|  | Blood (10%) | Hemoglobin subunit alpha OS=Homo sapiens | 8 | 246 | 2.8e-008 |
|  |  | Hemoglobin subunit beta OS=Homo sapiens | 12 | 227 | 3e-006 |
| S8 | Semen (90%) | Semenogelin-1 | 18 | 595 | 6.4e-007 |
|  |  | Semenogelin-2 | 7 | 272 | 6.1e-007 |
|  |  | Prostate-specific antigen | 2 | 99 | 5.2e-005 |
|  | Vaginal fluid (10%) | / | / | / | / |
| S9 | Vaginal fluid (90%) | Cornulin | 6 | 435 | 2.1e-011 |
|  |  | Cornifin-B | 1 | 48 | 0.011 |
|  | Blood (10%) | Hemoglobin subunit beta OS=Homo sapiens | 18 | 598 | 2.9e-009 |
|  |  | Hemoglobin subunit alpha OS=Homo sapiens | 11 | 443 | 1.1e-008 |
| S10 | Semen (90%) | Semenogelin-1 | 13 | 371 | 5.1e-007 |
|  |  | Semenogelin-2 | 9 | 288 | 8.2e-005 |
|  | Blood (10%) | Hemoglobin subunit delta OS=Homo sapiens | 4 | 140 | 7.8e-005 |
|  |  | Hemoglobin subunit beta OS=Homo sapiens | 4 | 137 | 1.1e-005 |
| S11 | Semen (40%) | Semenogelin-2 | 10 | 351 | 2.1e-006 |
|  |  | Semenogelin-1 | 9 | 284 | 3.6e-008 |
|  |  | Prostatic acid phosphatase | 5 | 222 | 2.8e-007 |
|  |  | Prostate-specific antigen | 3 | 92 | 5e-006 |
|  | Blood (40%) | Hemoglobin subunit beta OS=Homo sapiens | 56 | 1535 | 1.4e-011 |
|  |  | Hemoglobin subunit alpha OS=Pan troglodytes | 28 | 1053 | 1.9e-009 |
|  |  | Hemoglobin subunit delta OS=Homo sapiens | 26 | 1014 | 2.5e-006 |
|  | Saliva (20%) | Alpha-amylase | 1 | 75 | 2.1e-005 |
